# Production of the Allergenic Protein Alt a 1 by *Alternaria* Isolates from Working Environments

**DOI:** 10.3390/ijerph120202164

**Published:** 2015-02-16

**Authors:** Justyna Skóra, Anna Otlewska, Beata Gutarowska, Joanna Leszczyńska, Iwona Majak, Łukasz Stępień

**Affiliations:** 1 Institute of Fermentation Technology and Microbiology, Lodz University of Technology, 171/173 Wólczańska Str, 90-924 Łódź, Poland; E-Mails: anna.otlewska@p.lodz.pl (A.O.); beata.gutarowska@p.lodz.pl (B.G.); 2 Institute of General Food Chemistry, Lodz University of Technology, 171/173 Wólczańska Str, 90-924 Łódź, Poland; E-Mails: joanna.leszczynska@p.lodz.pl (J.L.); iwona.majak@gmail.com (I.M.); 3 Institute of Plant Genetics, Polish Academy of Sciences, 34 Strzeszyńska Str, 60-479 Poznań, Poland; E-Mail: lste@igr.poznan.pl

**Keywords:** *Alternaria*, workplaces, Alt a 1 protein, *Alt a 1* gene, ELISA

## Abstract

The aim of the study was to evaluate the ability of *Alternaria* isolates from workplaces to produce Alt a 1 allergenic protein, and to analyze whether technical materials (cellulose, compost, leather) present within the working environment stimulate or inhibit Alt a 1 production (ELISA test). Studies included identification of the isolated molds by nucleotide sequences analyzing of the ITS1/ITS2 regions, actin, calmodulin and *Alt a* 1 genes. It has been shown that *Alternaria* molds are significant part of microbiocenosis in the archive, museum, library, composting plant and tannery (14%–16% frequency in the air). The presence of the gene encoding the Alt a 1 protein has been detected for the strains: *Alternaria alternata*, *A. lini*, *A. limoniasperae A. nobilis* and *A. tenuissima.* Environmental strains produced Alt a 1 at higher concentrations (1.103–6.528 ng/mL) than a ATCC strain (0.551–0.975 ng/mL). It has been shown that the homogenization of the mycelium and the use of ultrafiltration allow a considerable increase of Alt a 1 concentration. Variations in the production of Alt a 1 protein, depend on the strain and extraction methods. These studies revealed no impact of the technical material from the workplaces on the production of Alt a 1 protein.

## 1. Introduction

Mold allergies affect from 3% to 40% of the human population, and this level changes depending on the country, region, sex, age and other factors [1]. So far, 78 allergens isolated from 18 mold species have been characterized in terms of chemical structure, nucleotide sequence and cellular function (International Union of Immunological Societies (WHO/IUIS) Allergen Nomenclature Sub-committee). These 18 species belong to the following genera: *Alternaria, Cladosporium, Penicillium, Aspergillus, Curvularia, Epicoccum, Stachybotrys* and *Trichophyton*. Previous work found that *Alternaria alternata* is the most allergenic species amongst those studied to date [2,3,4,5]. The high allergenicity of this species is hypothesized to be due to morphological changes in the respiratory epithelium, which is a result of the proteolytic enzymes produced by this mold. It is estimated that the presence of 100 spores of *Alternaria alternata* in 1 m^3^ of air is the threshold concentration, at which symptoms of allergy occur in patients who are sensitized to this species [6].

The major allergen produced by *Alternaria alternata* is the glycoprotein Alt a 1, which has a molecular weight of 30 kDa (dimer). This glycoprotein migrates as two separate bands of 16.4 and 15.3 kDa under reducing conditions on SDS-PAGE, suggesting that the monomers are linked by a disulfide bond. Alt a 1 is detected in the cytoplasm of mold spores and mycelia [7]. Recent *X*-ray crystallography revealed that the protein has a unique β-barrel fold that is cysteine-linked. Its biological function in cells is still unknown [8]. Alt a 1 leads to IgE-mediated hypersensitivity in more than 95% of *Alternaria*-sensitized patients [5,8,9]. It should also be noted that the number of allergens in *A. alternate* extracts may range from 10 to 30, and a few allergens were present in nearly all extracts studied [10].

Elevated levels of airborne *A. alternata* intensify rhinitis symptoms in allergic individuals [3,11]. They are the most common asthma-causing molds and also increase the severity of the disorder [1,12,13,14]. Long-term inhalation of antigens can cause allergic alveolitis (hypersensitivity pneumonitis (HP) [15,16]. As a source of allergens, molds of the *Alternaria* genus may also be a factor in causing asthma in bakers [17], and allergic alveolitis in carpenters and wood processors [18]. Other professional groups exposed to *A. alternata* allergens are workers in contact with either plant raw materials infested by these molds (grains, fruits, vegetables) or technical materials susceptible to microbial degradation (wood, paper, cloth). The high-risk groups that are in contact with the allergen include farmers, gardeners, employees of grain elevators, and food and herbal industries, forest service, monument conservators, librarians, museum personnel or those who deal with the storage and processing of municipal waste [18,19,20,21].

Occupational allergies associated with exposure to molds are poorly understood and difficult to detect. This is due to the multiplicity of factors arising from both occupational and non-occupational environments, which can cause symptoms similar to the disease [21]. Phylogenetic classification of the *Alternaria* genus is challenging. Recent molecular studies have revealed multiple non-monophyletic genera within the *Alternaria* complex and *Alternaria* species clades, which do not always correlate with morphological characteristic-based species groups [22]. In addition, the sequences of DNA fragments of identified strains show high homology (99%–100%) to multiple reference sequences (deposited in databases (e.g., GenBank)) at the same time and those derived from molds belonging to different species. Furthermore, Balajee *et al*. found that 20% of the ITS region sequence has come from erroneously identified mold species, and 14% of sequences derived from strains of the *Alternaria* genus, are incorrectly described [23].

The aim of this study was to evaluate the ability of environmental isolates of *Alternaria*, obtained from workplaces within libraries, a museum, a composting plant and a tannery, to produce the allergen, Alt a 1. The study also aimed to determine whether materials such as cellulose, compost and wet blue leather, that are stored/processed in the above working environments, stimulate or inhibit the amount of Alt a 1 generated.

The scope of the study included: (1) the isolation of *Alternaria* sp. strains from workplaces within libraries, a museum, a composting plant and a tannery; (2) determination of their percentages and frequencies of occurrence at each workplace; (3) morphological and genetic identification of the isolated *Alternaria* molds by analyzing nucleotide sequences of the ITS1/ITS2 regions, actin, calmodulin and *Alt a 1* genes; (4) confirming the presence of the gene encoding the Alt a 1 allergenic protein; (5) evaluating Alt a 1 production using an immunoassay; and (6) comparing the amounts of Alt a 1 from control media and from those simulating the environments from which the tested strains were isolated.

## 2. Materials and Methods

### 2.1. Working Environments

Quantitative analysis of molds were undertaken in the following working environments: archive (*N* = 1), museum (*N* = 1), libraries (*N* = 2), tannery (*N* = 1) and composting plant (*N* = 1). Descriptions of the working environments, and the number of air and surface samples are given in Table 1.

Airborne molds were isolated using an MAS-100 Eco Air Sampler (Merck, Darmstadt, Germany) according to the PN-EN 13098:2007 standard. Fifty and 100 L air samples were taken on DG18 agar medium (Dichloran Glicerol Selective Medium, Merck) and MEA medium (Malt Extract Agar, Merck) with chloramphenicol (0.1%) for determining total fungal number (including xerophilic and hydrophilic molds). Samples from surfaces (production surfaces, machinery and equipment) were collected using Replicate Organism Detection And Counting (RODAC) Envirocheck^®^ plates (Merck) containing Sabouraud medium (Merck).

The samples were incubated at 27 ± 2 °C for 5 days. Following incubation colonies were counted, and the results were expressed in CFU/m^3^ (air) or CFU/100 cm^2^ (surfaces). The proportion of each *Alternaria* isolate in the pool of molds was determined, and the incidence of airborne and surface molds in each workplace was measured.

**Table 1:** Characteristic working environmentals.

Isolation Working Environment	Description of Working Environment	Place of Samples Collection	
		Air	Surfaces
Archive (*N* = 12; *n* = 6)	Institution stores files, maps and books from 19th century factories, court records from 19 the 20th centuries no signs of moisture or molds.	Samples from the air were taken between shelves and stored objects	Samples from surfaces were collected from furniture, walls, stored objects.
Tannery (*N* = 18; *n* = 9)	Retannage and finishing of wet blue leather plant, short-term storage of palettes of raw material, vacuum drying of hides, movement of hides using hoists.	Air samples were collected next to the palette of wet blue hides, next to the vacuum drying oven, next to the racks with dried leather, in the tanned leather warehouse.	Samples from surfaces were collected from stored and processing leather and production machines.
Composting plant (*N* = 18; *n* = 9)	Green waste composting plant located in open area.	Samples were taken from the waste storage area, from the site of a fresh composting pile, near a compost pile that was turned 4 times, from the area around composting piles, while workers were selecting composting materials and building a new composting pile.	Samples from surfaces were collected from production machines.
Library A (*N* = 12; *n* = 6)	Institution located in basement. Inside—wooden bookshelves; lack of ventilation, signs of water damage on the walls, flaky paint, destroyed by molds book on the floor.	Samples of the air were taken between shelves and stored objects.	Samples from surfaces were collected from furniture, walls, stored objects.
Library B (*N* = 12; *n* = 6)	Institution stores books from 19–20th centuries; no signs of moisture or molds.	Samples were taken near metal shelves with books.	Samples from surfaces were collected from furniture, walls, stored objects.
Museum (*N* = 24; *n* = 12)	Collects machines (wood. steel) for processing of fibers mainly cotton and linen, textile products.	Samples of the air were taken near stored objects.	Samples from surfaces were collected from furniture, walls, stored objects.

*N*—number of air samples; *n*—number of samples from surfaces.

### 2.2. Identification of Tested Molds Strains

All mold isolates were characterized based on the macroscopic and microscopic characteristics of their colonies. They were then grouped into strains and identified using taxonomic keys after culturing them on MEA and Czapek–Dox Agar (Difco, Detroit, MI, USA) media, [24,25,26,27,28].

Genomic DNAs of all mold strains were extracted using the FastDNA Spin Kit for Soil (MP Biomedicals, Solon, OH, USA) following the manufacturer’s protocol, except for some modifications to the first step: samples were homogenized twice for 1 min, with one intervening minute on ice, instead of only once for 40 s. PCR was performed using an MJ Mini Gradient Thermal Cycler (Bio-Rad, Hercules, CA, USA). Universal primers ITS1 and ITS4 were used for the amplification of the internal transcribed spacer regions (ITS1/ITS2) [29]. The amplification of actin and calmodulin gene fragments was performed with primers Act-for, Act-rev and Calm-for, Calm-rev described by Lawrence *et al*. [30]. Primer sequences used in this study are presented in Table 2. Each PCR reaction was carried out in 50 µL volume containing 40 pmol of each primer, 1.5 U of RedTaq ReadyMix DNA polymerase (Sigma-Aldrich, St. Louis, MO, USA), 20 ng of template DNA and made up to 50 µL with PCR grade water. PCR products were detected by 1% (*w/v*) agarose gel electrophoresis in 0.5 × TBE buffer (Sigma-Aldrich).

**Table 2:** Sequences of primers used in this study.

Amplified Sequence	Primer Name	Primer Sequence (5′ > 3′)	Reference
ITS1/ITS2 region	ITS1	TCCGTAGGTGAACCTGCGG	White *et al.*, 1990 [29]
	ITS4	TCCTCCGCTTATTGATATGC	
Actin gene	Act-for	ATACCGGGGTACATGGTGG	Lawrence *et al.*, 2013 [30]
	Act-rev	TTCGGGTATGTGCAAGGC	
Calmodulin gene	Calm-for	AGCAAGTCTCCGAGTTCAAGG	Lawrence *et al.*, 2013 [30]
	Calm-rev	CTTCTGCATCATCAYCTGGACG	
*Alt a 1* gene	Alt-for	ATGCAGTTCACCACCATCGC	Hong *et al.* 2005 [31]
	Alt-rev	ACGAGGGTGAYGTAGGCGTC	

#### 2.2.1. DNA Sequencing and Sequence Analysis

PCR products were purified and nucleotide sequences of genes were obtained using the BigDye Terminator Ready Reaction Cycle Sequencing kit (Applied Biosystems, Foster City, CA, USA). The reaction products were analyzed using an Applied Biosystems model 3730 Genetic Analyzer at Genomed S.A. (Warsaw, Poland). 

The nucleotide sequences of ITS1/ITS2 regions, actin and calmodulin genes were proofread, assembled and aligned in Vector NTI Express Software (Life Technologies, Thermo Fisher Scientific Inc., Waltham, MA, USA). They were then compared with sequences available in The National Center for Biotechnology Information (NCBI, Bethesda, MD, USA) using the blastn algorithm (BLASTN 2.2.30+) [32].

#### 2.2.2. Phylogenetic Analysis 

Phylogenetic relationships were inferred using the Neighbor-Joining method using the Molecular Evolutionary Genetic Analysis (MEGA) Software, version 6.0 (MEGA, Tempe, AZ, USA) [33]. The evolutionary distances were computed using the Kimura 2-parameter method and are in the units of the number of base substitutions per site [34]. The analysis involved 74 nucleotide sequences of actin genes from the NCBI database. All reconstructions were tested by bootstrapping with 1000 replicates. All positions containing gaps and missing data were eliminated. There were a total of 931 positions in the final dataset.

### 2.3. Characteristics of the Tested Alternaria Strains

Seven isolates of *Alternaria* sp. from air or surfaces from the working environments (archive: one strain, museum: one strain, libraries: three strains, tannery: one strain and composting plant: one strain), were used to determine allergenic protein Alt a 1. In addition, *Alternaria alternata* ATCC 6663 (American Type Culture Collection, Manassas, VA, USA) was also used for Alt a 1 protein determination. Environmental strains were deposited into the Collection of Pure Culture LOCK CPC. The nucleotide sequences of the ITS1/ITS2 regions, actin and calmodulin genes obtained for the tested molds, were deposited in the NCBI GenBank database. Characteristics of the tested strains and the GenBank accessions numbers are given in Table 3.

#### Cultivation of Tested *Alternaria* Strains for Analysis of Alt a 1 Allergenic Protein

*Alternaria* strains were grown on a Czapek broth medium (Difco), and mineral M_0_ medium (glucose 5 g, MgSO_4_ × 7H_2_O 5 g, (NH_4_)_2_SO_4_ 3 g, KH_2_PO_4_ 1 g, yeast extract 10 g, distilled water to 1000 mL, pH 7.0) and supplemented mineral M_0_ medium with: cellulose 50 g compost extract 500 mL fragmented chrome-tanned leather (wet-blue leather shavings) 50 g. The compost extract was prepared by suspending 10 g of compost in 100 mL of distilled water, shaking the suspension for 30 min followed by vacuum filtration. Molds were cultured on media following the addition of a compound taken from the working environment, depending on the place where the strain was isolated (strains isolated from archives and libraries were grown on a medium containing cellulose, the tannery strain on a medium containing leather, while the strain from the composting plants on a medium with compost extract. Concentration of each compounds in medium was 5%. Using the mineral M_0_ medium containing the above additives allowed us to study the impact that compounds present within the working environments had on the production of allergenic protein. The samples were inoculated using a standardized inoculum (10^3^ spores/mL) and incubated at 27 ± 2 °C for 24 days in stationary conditions.

**Table 3:** Identification and characteristic of tested *Alternaria* strains.

Strain No.	Isolation Source	Identification	Accession Number ^*^	GenBank Accession Number ^**^		
				Actin Gene	Calmodulin Gene	ITS1/ITS2 Region
1	Archive/air	*Alternaria lini*	LOCK CPC 0610	KP341673	KP341681	KP341696
2	Tannery/air	*Alternaria nobilis*	LOCK CPC 0611	KP341674	KP341682	KP341697
3	Composting plant/air	*Alternaria limoniasperae*	LOCK CPC 0612	KP341675	KP341683	KP341698
4	Library A/ air	*Alternaria tenuissima*	LOCK CPC 0613	KP341676	KP341684	KP341699
5	Museum/surfaces of wooden washing machine	*Alternaria limoniasperae*	LOCK CPC 0614	KP341677	KP341685	KP341700
6	Library B/air	*Alternaria lini*	LOCK CPC 0615	KP341678	KP341686	KP341701
7	Library B/surfaces of book cover	*Alternaria lini*	LOCK CPC 0616	KP341679	KP341687	KP341702
8	*Alternaria alternata* ATCC 6663			KP341672	KP341680	KP341703

^*^ deposited in The Lock Collection of Pure Culture (Lodz, Poland); ^**^ deposited in the National Center for Biotechnology Information GenBank database.

In the first stage of the study, methods for obtaining Alt a 1 were compared between a selected environmental strain (strain no. 7—*Alternaria lini*) and *Alternaria alternata* ATCC 6663. For this purpose, the molds were grown on CYA medium using the conditions described above, and three types of extractions were used: (1) filtered (0.22 μm pore diameter membrane, Millipore, Bedford, MA, USA), (2) homogenized (4 min, 35,000 rpm, homogenizer BioGen PRO 200; PROScientific, Oxford, CT, USA) and filtered (0.22 μm pore diameter membrane, Millipore), (3), homogenized (4 min, 35,000 rpm, homogenizer BioGen PRO 200; PROScientific), filtered (standard qualitative filter, Whatman), dialyzed by ultrafiltration (Pellicon system, Millipore) with a 10,000 Da cut-off point.

Method 2 (which used homogenization and extract filtration) was selected for the second stage of the study. This method was used to assess the level of Alt a 1 on each culture medium (CYA, M_0_ and supplemented M_0_). Using the same method, media, which were not inoculated, were also filtered and used as controls. Three repetitions for each sample were performed.

### 2.4. Assessment of Alt a 1 Production by Alternaria Strains

#### 2.4.1. Detection and Identification of *Alt a 1* Gene

PCR reactions were undertaken to detect and identify the nucleotide sequences encoding the *Alt a 1* gene. The genomic DNAs isolated from mold strains were used as templates. The amplification was carried out in a total volume of 50 µL using the Alt-for and Alt-rev (Table 1) primers, described by Hong *et al*. [31]. PCR products were analyzed by electrophoresis in 1% (*w/v*) agarose in 0.5 × TBE buffer (Sigma-Aldrich). DNA from *Alternaria alternata* ATCC 6663 were used as positive control for *Alt a 1* gene amplification.

#### 2.4.2. Alt a 1 Allergenic Protein Determination

Alt a 1 protein concentrations in the *Alternaria* culture filtrates were measured using a monoclonal antibody-based ELISA test (Indoor Biotechnologies Inc., Charlottesville, VA, USA), as described by Vailes *et al*. [35]. Primary mouse monoclonal IgG antibodies against rAlt a 1 antigen (recombinant clone 2C10 G4) at 1 µg/mL concentration were adsorbed on a microplate following incubation for 24 h at 4 °C. Subsequently, the plate was blocked with a 3% solution of defatted milk in PBS-0.05% Tween 20 (Sigma-Aldrich P3563). One hundred µL of each sample per well was transferred onto the plate. A standard curve was simultaneously prepared with rAlt a 1 standard protein. The secondary antibody was clone 3B6- a mouse monoclonal IgG antibody against Alt a 1, labeled with biotin. To develop the plate, streptavidin bound to phosphatase was added (Sigma-Aldrich S2890-1MG), and the reaction was performed with adequate amounts of substrate (pNPP; Sigma- Aldrich P7998-100mL). The results were recorded with a ThermoLabsystem RC reader (ThermoLabSystems, Helsinki, Finland) at 405 nm wavelength. Filtered media that were not inoculated were used as controls (CYA medium, M_0_ medium and M_0_ medium supplemented with cellulose, compost extract and wet-blue leather). Three repetitions were performed to determine Alt a 1 concentration.

#### 2.4.3. Statistical Analysis 

The differences between Alt a 1 concentrations from CYA and mineral medium, as well as from mineral and supplemented (cellulose, wet-blue leather, compost extract) mineral medium, were analyzed for all tested strains.

We also compared Alt a 1 concentrations from CYA medium, for *Alternaria lini* (strain no. 7) and *Alternaria alternata* ATCC 6663, that were obtained using the three tested methods (filtration, homogenization, ultrafiltration). Analysis was performed using One-Way Analysis of Variance (ANO­VA). Differences were considered significant at *p* < 0.05. The data were analyzed using the Origin 6.1 computer program (OriginLab Corporation, Northhampton, MA, USA).

## 3. Results

Air and surfaces in the studied workplaces were contaminated by molds to varying degrees. The highest numbers of airborne molds were recorded in Library A and the composting plant (5.3 × 10^3^ CFU/m^3^ and 1.7 × 10^3^ CFU/m^3^, respectively). The lowest airborne fungal contamination was detected in the Museum and Archive, and it ranged from 7.2 × 10^1^ to 9.2 × 10^1^ CFU/m^3^. Fungal contamination of surfaces varied by institution, and was mainly dependent on the hygienic conditions of the stored objects and equipment. This concentration ranged from 1.5 × 10^1^ CFU/100 cm^2^ to 3.3 ×10^3^ CFU/100 cm^2^ (Table 4).

**Table 4:** Mold contamination of tested workplaces.

Working Environment	Level of Fungal Contamination at Workplaces		*Alternaria* sp. Concentration					
	Air [CFU/m^3^]	Surfaces [CFU/100cm^2^]	Number		Percentage [%]		Frequency [%]	
			Air [CFU/m^3^]	Surfaces [CFU/100cm^2^]	Air	Surfaces	Air	Surfaces
Archive	M: 9.2 × 10^1^	M: 3.3 × 10^3^	6.1 × 10^0^	0.0	6.6	0.0	14.3	0.0
	Min: 1.0 × 10^1^	Min: 5.0 × 10^0^						
	Max: 2.1 × 10^2^	Max: 1.2 × 10^4^						
	SD: 7.9 × 10^1^	SD: 1.2 × 10^3^						
Tannery	M:7.3 × 10^2^	M:1.5 × 10^1^	7.3 × 10^1^	0.0	10.0	0.0	39.0	0.0
	Min:1.7 × 10^2^	Min 5.1 × 10^0^						
	Max:2.21 × 0^3^	Max:4.6 × 10^1^						
	SD:3.0 × 10^2^	SD:1.5 × 10^1^						
Composting plant	M:1.7 × 10^3^	M:1.5 × 10^3^	1.0 × 10^2^	0.0	5.9	0.0	64.0	0.0
	Min:8.8 × 10^2^	Min:1.0 × 10^3^						
	Max:3.4 × 10^3^	Max:2.0 × 10^3^						
	SD:7.2 × 10^2^	SD:5.1 × 10^2^						
Library A	M: 5.3 × 10^3^	M: 1.3 × 10^2^	2.9 × 10^2^	0.0	5.5	0.0	16.7	0.0
	Min: 2.2 × 10^3^	Min: 4.2 × 10^0^						
	Max: 1.1 × 10^4^	Max: 8.4 × 10^2^						
	SD: 2.9 × 10^3^	SD: 2.1 × 10^2^						
Library B	M: 5.2 × 10^2^	M: 1.0 × 10^3^	1.0 × 10^1^	2.1 × 10^2^	1.9	21.5	33.3	50.0
	Min: 2.5 × 10^2^	Min: 5.0 × 10°						
	Max: 1.3 × 10^3^	Max: 6.4 × 10^3^						
	SD: 4.4 × 10^2^	SD: 3.9 × 10^3^						
Museum	M: 7.7 × 10^1^	M: 1.0 × 10^2^	0.0	4.9 × 10^1^	0.0	49.0	0.0	14.3
	Min.: 0.0	Min.: 0.0						
	Max.:2.4 × 10^2^	Maks.: 8.0 × 10^2^						
	SD: 5.8 × 10^1^	SD: 2.2 × 10^2^						

M—arithmetic mean; Min/Max-minimum/maximum value; SD- standard deviation.

Molds of the *Alternaria* genus were present in the air of the workplaces tested, (strains No. 1–7) at concentrations ranging from 6.1 × 10^0^ CFU/m^3^ to 2.9 × 10^2^ CFU/m^3^, accounting for 2%–10% of the fungal aerosols in the analyzed environments, and were detected using culture methods. *Alternaria* strains were found on surfaces in Museum and Library B at concentrations of 4.9 × 10^1^ CFU/100 cm^2^ to 2.1 × 10^2^ CFU/100 cm^2^, representing 9%–49% of all molds. *Alternaria* strains were isolated from the workplaces at a high incidence: 14%–64% in the air and 14%–50% on surfaces (Table 4).

**Figure 1:**
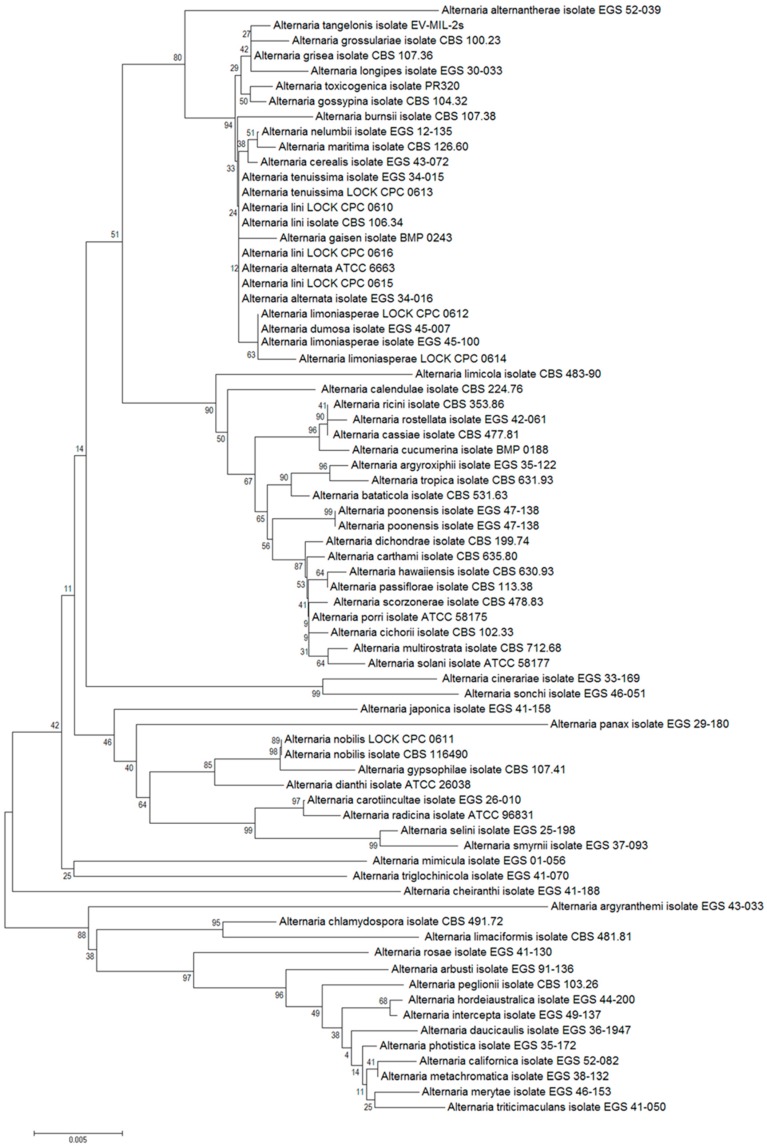
The phylogenetic tree constructed on the basis of actin gene sequences of reference and tested *Alternaria* strains. The tree was constructed using the Neighbor-Joining method and tested by bootstrapping (1000 replicates).

Genetic identification based on nucleotide sequence analyses of the ITS1/ITS2 regions, actin, calmodulin and the *Alt a 1* gene confirmed the phylogenetic affiliation of the three tested fungal strains with *Alternaria lini*, two with *A. limoniasperae*, while the remaining strains were affiliated with *A. tenuissima*, *A. nobilis* and *A. alternata*. The degree of similarity of all analyzed nucleotide sequences ranged from 87% to 99% (Table 5, Figure 1).

**Table 5:** Detection of *Alt a 1* gene for tested *Alternaria* strains.

Strain No.	Strain	Presence of *Alt a 1* gene	GenBank accession number of *Alt a 1* gene ^*^
1	*Alternaria lini*	+	KP341689
2	*Alternaria nobilis*	+	KP341690
3	*Alternaria limoniasperae*	+	KP341691
4	*Alternaria tenuissima*	+	KP341692
5	*Alternaria limoniasperae*	+	KP341693
6	*Alternaria lini*	+	KP341694
7	*Alternaria lini*	+	KP341695
8	*Alternaria alternata* ATCC 6663	+	KP341688	

(+) – presence; ^*^ deposited in the National Center for Biotechnology Information GenBank database.

**Figure 2:**
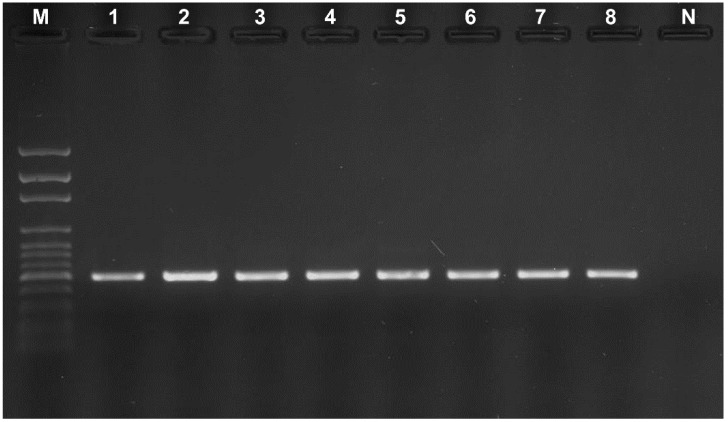
Detection of *Alt a 1* gene in tested *Alternaria* strains. M—molecular weight marker from 100 bp to 3000 bp (Solis BioDyne, Tartu, Estonia), 1–7—tested *Alternaria* strains, 8—*Alternaria alternata* ATCC 6663 (positive control), N—negative control.

PCR amplification of the *Alt a 1* gene resulted in products that were approximately 510 bp in length. A gene encoding the allergenic protein Alt a 1 was identified in the tested strains, i.e. the isolates from the studied workplaces, and *Alternaria alternata* from ATCC (Figure 2).

Alt a 1 levels obtained on CYA culture medium were compared after culturing a selected environmental strain (*Alternaria lini*) and *Alternaria alternata* ATCC 6663, using different methods of sample preparation: filtration (F), filtration preceded by mycelium homogenization (HF) and sample concentration using ultrafiltration (HFU) (Table 6). Alt a 1 levels increased from 0.446–0.902 ng/mL to 1.072–1.303 ng/mL following homogenization compared to its concentration in the post-culture filtrate. However, the highest (*p* < 0.05) protein levels ranging from 8.932–44.071 ng/mL were obtained after using ultrafiltration for sample concentration (Table 6).

**Table 6:** Comparison of methods of Alt a 1 protein obtaining for selected strains of *Alternaria*.

Species	Concentration of Alt a 1 in ELISA Test [ng/mL]		
	F	HF	HFU
*Alternaria lini*	0.902 ± 0.039	1.303 ± 0.277	8.932 ± 0.362 ^*^^,$^
*Alternaria alternata* ATCC 6663	0.446 ± 0.022	1.072 ± 0.046 #	44.071 ± 7.096 ^*^^,$^

F—filtration. FH—homogenization and filtration. HU—homogenization, filtration and ultrafiltration. # significantly difference between Alt a 1 content obtained from F and HF methods (One-Way ANOVA, *p* < 0.05); ^*^ significantly difference between Alt a 1 content obtained from F and HFU methods (One-Way ANOVA, *p* < 0.05); ^$^ significantly difference between Alt a 1 content obtained from HF and HFU methods (One-Way ANOVA, *p* < 0.05).

Strains isolated from workplaces produced Alt a 1 in concentrations ranging from 1.103 to 6.528 ng/mL, depending on the strain and culture medium—CYA, M_0_ or supplemented M_0_ (Table 7).

**Table 7:** Production Alt a 1 allergenic protein by tested *Alternaria* strains.

Strain No.	Species	Concentration of Alt a 1 in ELISA test [ng/mL]		
		CYA	M_0_	Suplemented M_0_
1	*Alternaria lini*	6.528 ± 2.219	1.938 ± 0.187 ^*^^↓^	C: 1.627 ± 0.082
2	*Alternaria nobilis*	1.890 ± 0.269	1.103 ± 0.484	W: 2.444 ± 1.411
3	*Alternaria limoniasperae*	1.500 ± 0.201	3.066 ± 0.937 ^*^^↑^	CE: 2.051 ± 0.835
4	*Alternaria tenuissima*	2.010 ± 0.373	4.110 ± 0.638 ^*^^↑^	C: 2.508 ± 0.800
5	*Alternaria limoniasperae*	2.598 ± 1.694	5.164 ± 3.231	C: 1.584 ± 0.644
6	*Alternaria lini*	3.260 ± 0.437	1.680 ± 0.494 ^*^^↓^	C: 1.126 ± 0.677
7	*Alternaria lini*	1.485 ± 1.010	2.296 ± 0.995	C: 1.572 ± 0.401
8	*Alternaria alternata* ATCC 6663	0.902 ± 0.299	0.824 ± 0.280	C: 0.598 ± 0.077
				W: 0.975 ± 0.430
				CE: 0.551 ± 0.102

C—cellulose; W—wet-blue leather; CE—compost extract; ^*^ significantly difference between Alt a 1 concentration from CYA medium and M_0_ medium (One-Way ANOVA, *p* < 0.05); ^↑^—increase; ^↓^—decrease.

There was no statistically significant relationship between the type of medium and the concentration of Alt a 1 produced by the tested strains on CYA medium and mineral M_0_ medium. In the case of two strains (*A. limoniasperae* isolated from the composting plant and *A. tenuissima* from the library), mineral M_0_ medium stimulated the molds to produce more allergen compared to CYA medium. A different situation was observed for two strains of *Alternaria lini* isolated from the archive and the library. A lower, statistically significant, Alt a 1 level was achieved on M_0_ medium compared to its concentration on CYA medium. There were no statistically significant differences in Alt a 1 levels obtained on mineral M_0_ medium and on the medium simulating environmental conditions. It was shown that the addition of material (cellulose, compost, wet-blue leather) to M_0_ culture medium did not affect the concentration of Alt a 1. The culture collection strain *Alternaria alternata* ATCC 6663 produced smaller amounts of the allergen (0.551–0.975 ng/mL), compared to the environmental isolates (Table 7).

## 4. Discussion 

The highest concentration of airborne molds amongst the workplaces was found in the composting plant, slightly lower in the libraries and the tannery, and lowest in the museum and the archive. However, fungal contamination levels in all studied workplaces (from 7.7 × 10^1^ to 5.3 × 10^3^ CFU/m^3^) were lower than the threshold values of occupational exposure specified by the Polish Committee (for the Highest Permissible Concentrations and Intensities of Noxious Agents at the Workplace: 5.0 × 10^3^/5.0 × 10^4^ CFU/m^3^ for the total fungal count in public facilities/organic dust contaminated workplaces) [36]. However, there are no (national or international) legal regulations establishing permissible workplace concentrations of number of microorganisms [37]. Evaluation of the level of airborne microbiological contamination in working environments is currently carried out on the basis of recommendations from the literature including “threshold limit value”, “acceptable maximum value”; “maximum allowable concentration” *etc*. [38,39]. What is worth emphasizing a series of studies including various fungal species suggest that respiratory symptoms, airway inflammation, and lung function impairment begin to related with exposure levels of approximately 10^5^ spores/m^3^ [40]. This contamination level is probably too high if spores from mycotoxin-producing and/or opportunistic pathogenic species are prevalent. Moreover, people with asthma and sensitized to fungal allergens are more susceptible than working population in general. It is estimated that *Alternaria alternata* spores in concentration 100/m^3^ in the air can cause allergy symptoms in sensitized people [6]. In 2 from 6 tested working environment (composting plant and Library A) this limit has been exceeded in case of *Alternaria* strains. Employees of these workplaces are the most exposed to allergic diseases.

The present study shows that molds of the genus *Alternaria*, producing the allergenic protein Alt a 1, represent a significant component of the ecosystem of the tested workplaces. This is based on their relative proportions (2%–49%), and their frequencies of isolation from the pool of all detected molds (14%–64%). Species of the genus *Alternaria* were already isolated from workplaces such as libraries, archives and museums in previous studies [41,42]. Grisoni *et al*. detected high levels of *Alternaria alternata* in the vicinity of a composting plant and a wastewater treatment plant throughout the year, and this concentration decreased with increasing distance from the plants [43].

The present study indicates that persons employed in museums, archives, libraries, composting plants and tanneries involve a risk of mould exposure of the *Alternaria* genus. The ability of the isolates to produce Alt a 1 protein has been confirmed. Other authors have already pointed to the allergenic effects of *Alternaria alternata* in the home and workplace environments, especially on people exposed to moldy wood dust, as well as on bakers and museum personnel [17,21,44].

Also should be mentioned that *Alternaria* like other molds is a potential sorce of β-D-glucan (a polymer of N-acetyl-β-D-glucosamine, chitin). β-D-Glucan is a component of the fungal cell wall, which is a potent activator of the immune system, causing a non-allergic respiratory disease. The effects of exposure to glucan can lead to fatigue, headaches and other neurological symptoms in exposed workers [45,46]. Occupational exposure to β-D-glucan has been most commonly described in the municipal waste industry, wastewater treatment plants and in different branches of agriculture [47,48,49].

In the future, the sensitivity of employees within tested environments to Alt a 1 should be confirmed by medical examination. A occupational risk assessment at the workplace is the responsibility of the employer. Risk assessment should be done if there is a suspicion that the employee’s health problems are associated with exposure to a biological agent at the workplace. The suspicion that the disease results from the employee’s exposure to mold must be confirmed by an occupational physician. Diagnostics of the allergy include skin prick tests and determination of IgE specific for mold allergens of employees in the serum. Many authors claim that allergen extracts produced by molds, show a clear lack of homogeneity for a number of reasons, including strain identification and variability, culture conditions or production methods [50,51,52].

In this study, a gene encoding the major allergen Alt a 1 was detected not only in *Alternaria alternata* strains, but also in *A. lini*, *A. limoniasperae*, *A. tenuissima* and *A. nobilis*. Hong *et al*. found the *Alt a 1* gene and its homologs in *A. alternata A. limoniasperae, A. tenuissima* and others species of the genus *Alternaria* [31]. Moreover, a comparison between Alt a 1 homologs of several *Alternaria* species revealed greater sequence divergence than that found in similar comparisons of other ribosomal and protein-coding genes. Therefore it is used as a marker to identify strains belonging to this species.

Sáenz-de-Santamaría *et al*., suggested a strong cross-reactivity with other species affiliated with *Alternaria alternata*, indicating that it may also concern the genera *Stemphyllium*, *Ulocladium* or *Curvularia* [52]. This fact is very importand in assesment of allery risk in working environments. It should be be taken into account that detection of Alt a 1 sensivity in workers is not always related to *Alternaria alternata* sensitiveness.

In the present study, allergen concentrations obtained from the culture filtrates of *Alternaria* strains ranged from 0.598 to 6.528 ng/mL, which confirm Alt a 1 production variability. Sáenz-de-Santamaría *et al*., demonstrated that various species of the *Alternaria* genus produce different amounts of Alt a 1, e.g., *A. tenuisima* FMR5813 produces over four times more Alt a 1 than *A. alternata* IMMS 93039 [52]. Martínez *et al*., studied various strains of the *A. alternata* species, and Alt a 1 levels in post-culture extracts ranged from 0.5 to 16.1 ng/mL [53]. It is also important to note that environmental strains of *Alternaria alternata* produced higher levels of Alt a 1 (1.103 to 6.528 ng/mL) than the strain obtained from the ATCC collection (0.551 to 0.975 ng/mL).

So far, little is known about the impact that technical materials, present in the natural environments of molds, have on allergen production. This study could not demonstrate an effect of technical materials on the production of Alt a 1. For all strains tested, Alt a 1 levels in the mineral medium containing cellulose, compost or wet-blue leather, were statistically comparable to those obtained from the control medium (without additives).

Different results were obtained by Gutarowska *et al*., who studied molds isolated from home environments [54]. They found that more allergenic proteins were produced on building materials (wallpaper, carton-gypsum board), compared to laboratory medium M_0_. Other studies also reported the effect of different culture media on the amount of allergens produced by molds belonging to the *Aspergillus*, *Cladosporium* and *Penicillium* genera [55,56]. 

Alt a 1 levels are significantly affected by the method used to obtain proteins from a mold culture. In the present study, the allergenic protein Alt a 1 was extracted from a culture filtrate. Other studies also showed that Alt a 1 accumulates in culture media, and therefore culture filtrates constitute the optimum source of this protein [57,58,59]. Furthermore, the present study has demonstrated that the homogenization of the mycelium increases Alt a 1 levels by 44%–140%, while ultrafiltration increases it by 10%–98%. The results show that homogenization and ultrafiltration seem to be suitable methods for the industrial production of Alt a 1, for example, for immunotherapeutic purposes. Lizaso *et al.* found strong reactivity of *Alternaria alternata* extracts, prepared using *in vivo* ultrafiltration, in skin prick tests (SPT) [59].

## 5. Conclusions

Amongst the tested work environments the highest microbial contamination was found in composting plants, libraries and tanneries, while lower in museums and archives. Study demonstrated that molds from the genus *Alternaria* are an important part of microbiocenosis present in the tested workplaces as revealed on the basis of significant percentages and frequency of isolation amongst all fungi. The presence of the gene *Alt a 1* encoding the allergenic proteins Alt a 1 and the expression of this gene has been detected for strains of *Alternaria alternata*, *A. limoniasperae A. lini, A. nobilis* and *A. tenuissima.* Variations in the production of protein Alt a 1, depend on the strain and extraction methods. Environmental *Alternaria* strains produced Alt a 1 at higher concentrations (1.103 to 6.528 ng/mL) than a strain derived from ATCC collection (0.551–0.975 ng/mL). Study demonstrated that the homogenization of the mycelium and the use of ultrafiltration allow for a considerable increase of Alt a 1 concentration in the mold culture extract. These studies revealed no impact of the technical material coming from the workplaces from which strains were isolated (cellulose, compost, wet-blue leather) on the production of Alt a 1.
